# Supplementary figures and images for: Indole-3-Propionic Acid, a Functional Metabolite of Clostridium sporogenes, Promotes Muscle Tissue Development and Reduces Muscle Cell Inflammation
Source: Int J Mol Sci. 2021 Nov 18;22(22):12435. doi: 10.3390/ijms222212435 (PMC8619491; doi:10.3390/ijms222212435)

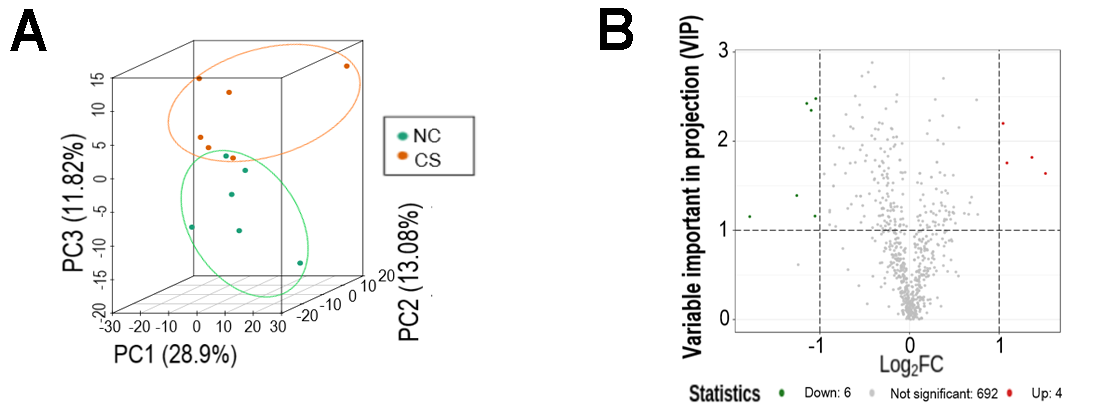

Supplement: Supplementary file 1 [file ijms-22-12435-s001.zip › Figure S1.tif]

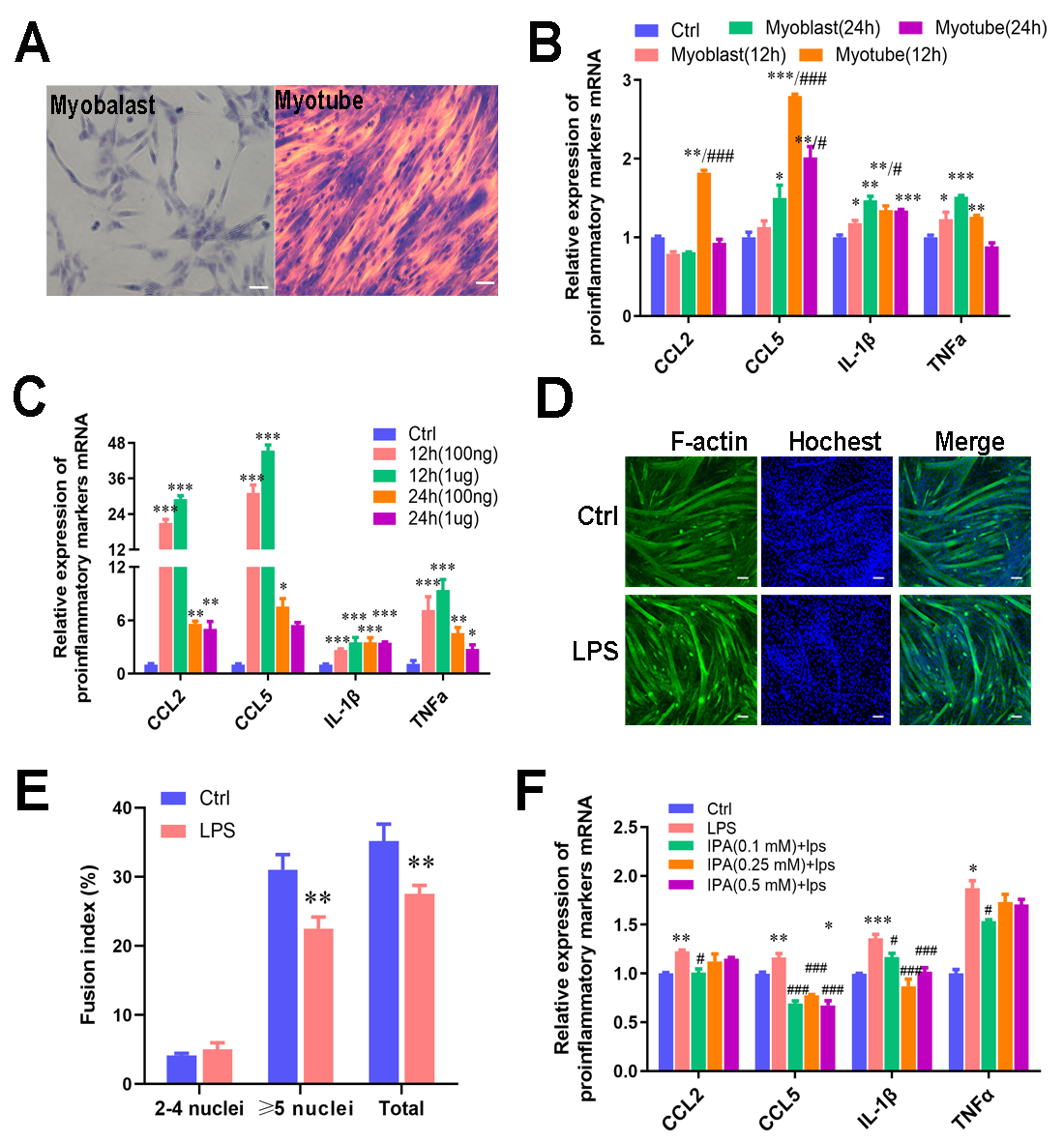

Supplement: Supplementary file 1 [file ijms-22-12435-s001.zip › Figure S2.tif]
